# Pulmonary embolism presenting as delirium: an acute confusional state in an elderly patient—a case report

**DOI:** 10.1186/s43162-023-00193-5

**Published:** 2023-02-06

**Authors:** Chidimma A. Ahaneku, Benard B. Akpu, Chibueze H. Njoku, David E. Elem, Bassey E. Ekeng

**Affiliations:** 1 Department of Internal Medicine, Respiratory Unit, University of Calabar, Teaching Hospital, Calabar, Nigeria; 2 Department of Internal Medicine, Cardiology, Unit, University of Calabar, Teaching Hospital, Calabar, Nigeria; 3 Department of Internal Medicine, Infectious Disease Unit, University of Calabar, Teaching Hospital, Calabar, Nigeria

**Keywords:** Acute confusional state, Delirium, D-dimer, High-resolution computed tomography, Pulmonary embolism

## Abstract

**Background:**

Large numbers of elderly patients are admitted to hospitals in acute confusional states. In many, the underlying causes are easily found; in some, correct diagnosis is difficult. Pulmonary embolism (PE), the most serious clinical presentation of venous thromboembolism, is often misdiagnosed because of its non-specific features including delirium.

**Case presentation:**

A 73-year-old woman was admitted to our hospital in a confused state with no obvious risk factors of PE. D-dimer levels were elevated and contrast-enhanced high-resolution computed tomography (HRCT) of the chest confirmed the diagnosis of PE. She was treated with enoxaparin and discharged on dabigatran. Her symptoms had resolved at the time of discharge, and she has been stable for over three month’s follow-up visit.

**Conclusion:**

PE should be regarded as a differential in elderly patients with an acute confusional state despite the absence of obvious risk factors. Investigating for and treating when confirmed may save a life.

## Background

PE, the most lethal presentation of venous thromboembolism, is defined as obstruction of the pulmonary arteries by thrombi from veins outside the lungs [1–3]. PE can also originate de novo without any evidence of DVT [4]. PE is one of the most common cardiovascular diseases, occurring in 1–2 per 1000 people annually [2].Virchow first described the process of thrombosis as a triad of stasis, hypercoagulability, and endothelial injury [5, 6]. PE risk factors include obesity, immobilization, cancer, surgery, trauma, pregnancy, oral contraceptives or hormone replacement therapies, prior history of PE, or known hype-coagulable disorder; however, 30% of patients with PE have no detectable provoking factors [2]. PE can mimic or co-exist with other medical conditions, thus presenting a diagnostic puzzle [7]. Patients may present in a classical way with shortness of breath, pleuritic chest pain, coughing, orthopnea, wheezing, hemoptysis or less commonly cardiac arrhythmias, syncopal attack or more devastatingly, and circulatory collapse [3, 8], depending on the size, number, site of thrombi/emboli, and risk factors present [7]. Atypical presentations of PE include back pain, syncope and acute confusion [9]. Acute confusion is common in the elderly, and cerebral hypoxia due to pneumonia or cardiac failure is a well-recognized cause [10]. The other common cause of hypoxia, PE, is clinically difficult to diagnose and the diagnosis “is missed more often than it is made,” or only found at post mortem because of its frequent atypical presentations [10, 11]. Prompt and accurate diagnosis of PE is facilitated by a clinical evaluation that assesses the probability of PE and makes appropriate use of the plasma D-dimer enzyme-linked immunosorbent assay (ELISA) and chest computed tomography (CT) scanning [12].

To the best of our knowledge, PE presenting as delirium is yet to be reported in Africa, including Nigeria.

A significant proportion of elderly patients who present with delirium may have undiagnosed PE as was the case in our patient. We report this case of delirium secondary to PE in a 73-year-old female who presented with features suggestive of other clinical entities.

### Case report

The patient was a previously fit, independent, non-hypertensive, and non-diabetic woman admitted at the University of Calabar Teaching Hospital with a week history of delirium. She was initially noticed to be unusually quiet, later restless and unable to sleep, with poor attention span, inability to follow a conversation, loss of appetite, and occipital headache. There was no weakness of any part of her body, no loss of consciousness, fall or trauma to the head, fever, neck pain, blurring of vision, slurred speech, or seizures.

On examination, she was restless, afebrile (temperature: 37℃), not pale, anicteric, and not cyanosed with no pedal edema. Glasgow Coma score was 14/15. She was confused and disorientated in time, place, and person with no focal neurological deficit or signs of a cerebellar disorder. The Mini-Mental State Examination (MMSE) score was 25/30. The pulse rate was 100 beats/minute. The blood pressure was 150/90 mmHg. The apex was displaced to the 6th left intercostal space, anterior axillary line. The heart sounds were S4, S1, and S2. The respiratory rate was 28 cycles per minute. Oxygen saturation (SPO_2_) was 91%. Breath sounds were decreased with coarse crepitation on the right lower lung zone. The abdominal examination was not remarkable.

**Fig. 1:**
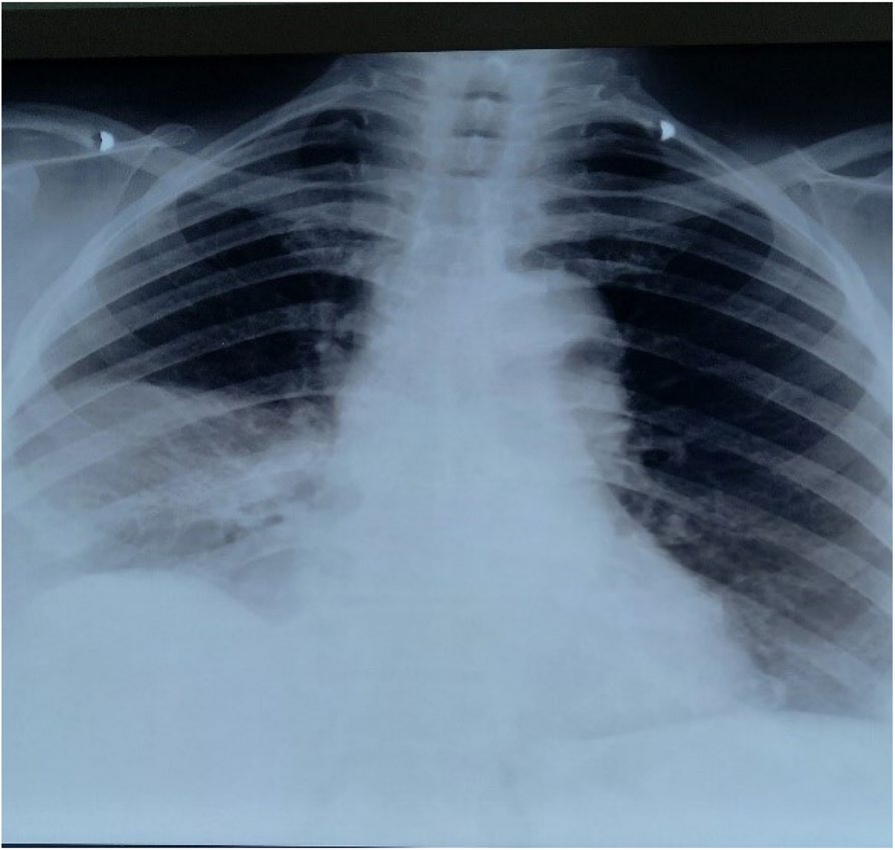
Chest X-ray showing homogeneous opacity on the right middle and lower lung zones and elevated right hemi-diaphragm

Serum electrolyte, urea and creatinine, and white cell and platelet counts were within the reference interval. Hemoglobin was slightly decreased (Table 1). The reverse transcription polymerase chain reaction (RT-PCR) test of a nasopharyngeal swab was negative for SARS-CoV-2. Arterial blood gas analysis was not done for a lack of equipment. The urine culture yielded no growth of pathogens. Electrocardiography showed sinus tachycardia while echocardiography was suggestive of hypertensive heart disease with diastolic dysfunction. The chest X-ray was suggestive of right lobar pneumonia (Fig. 1).

**Table 1:** Laboratory finding

Test	Value	Reference range
Hemoglobin (g/dl)	12.0	13.5–17.5
White-cell count (per mm^3^)	8200	4000–10,000
Platelet count (per mm^3^)	268,000	150,000–400,000
Urea (mmol/l)	5.5	2.6–6.7
Creatinine (umol/l)	100	53–106
Sodium (mmol/l)	136	135–144
Potassium (mmol/l)	4.8	3.5–5.0
INR (international normalized ratio)	1.3	< 1.1
D-dimer (ng/mL)	1902.5	< 500

**Fig. 2:**
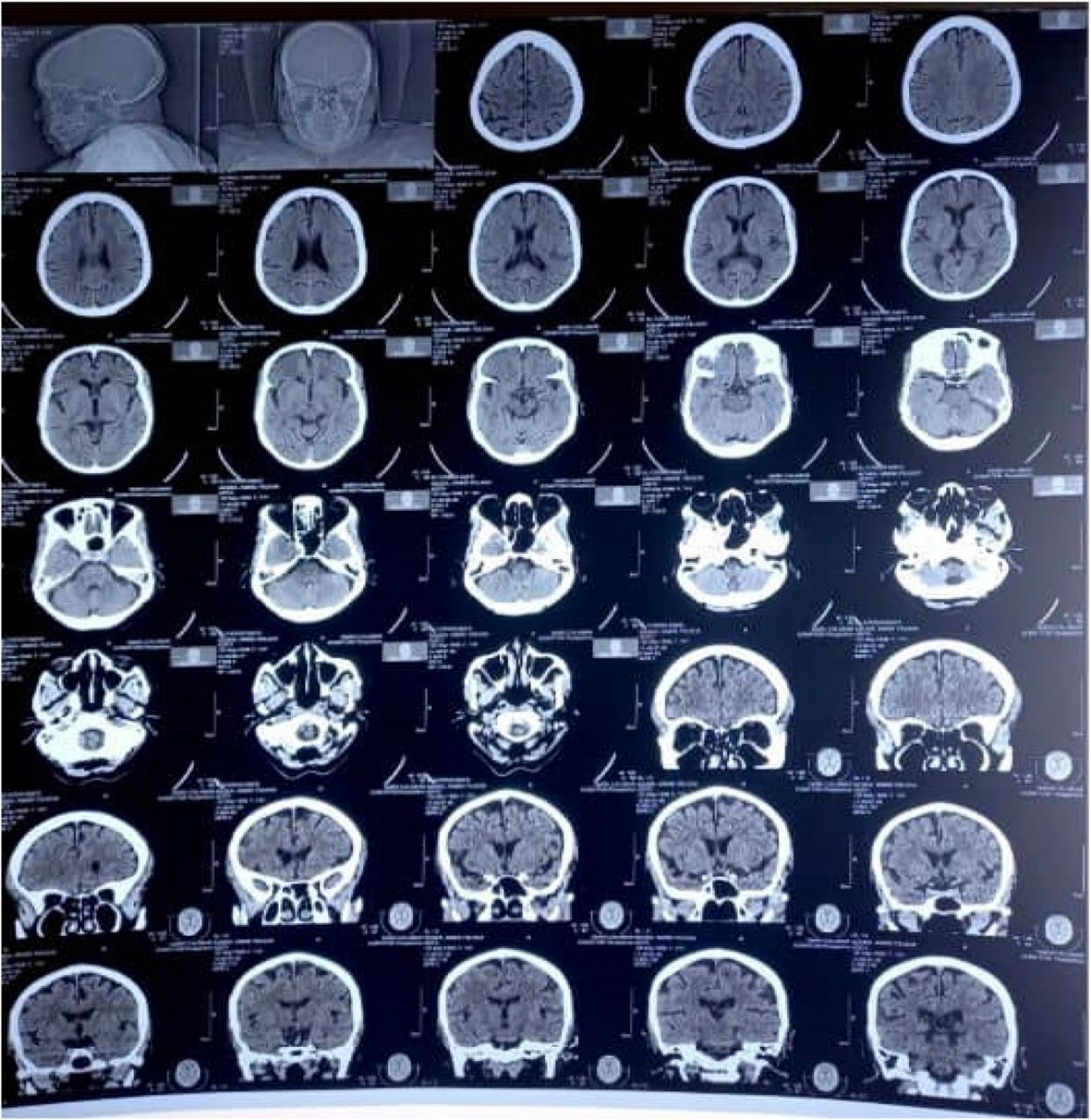
Cranial CT scan showing mild age-related cerebral atrophy, no focal intracerebral, or intracerebellar mass lesion seen

The cranial CT scan showed mild age-related cerebral atrophy, and no focal intracerebral or intracerebellar mass lesion or collection was seen (Fig. 2).

A diagnosis of delirium secondary to community-acquired pneumonia in a newly diagnosed hypertensive was made by the admitting physician. Oxygen by facemask at 4–6L/min was prescribed. Intravenous levofloxacin and tablet azithromycin were commenced for the treatment of the pneumonia. Tablets amlodipine and lisinopril were prescribed to control her hypertension. No remarkable improvement was noticed after 5 days. She remained confused and tachypneic with SPO_2_ fluctuating between 86 and 92%.

The pulmonology team was invited to review her because of suspicion of PE although there were no risk factors. The MMSE Score- 25/30 showed no cognitive impairment. Well’s score was low (1.5), but D-dimer was elevated (1902.5 ng/mL). Duplex Doppler ultrasonography of the lower limbs although requested was not done due to financial constraints.

**Fig. 3:**
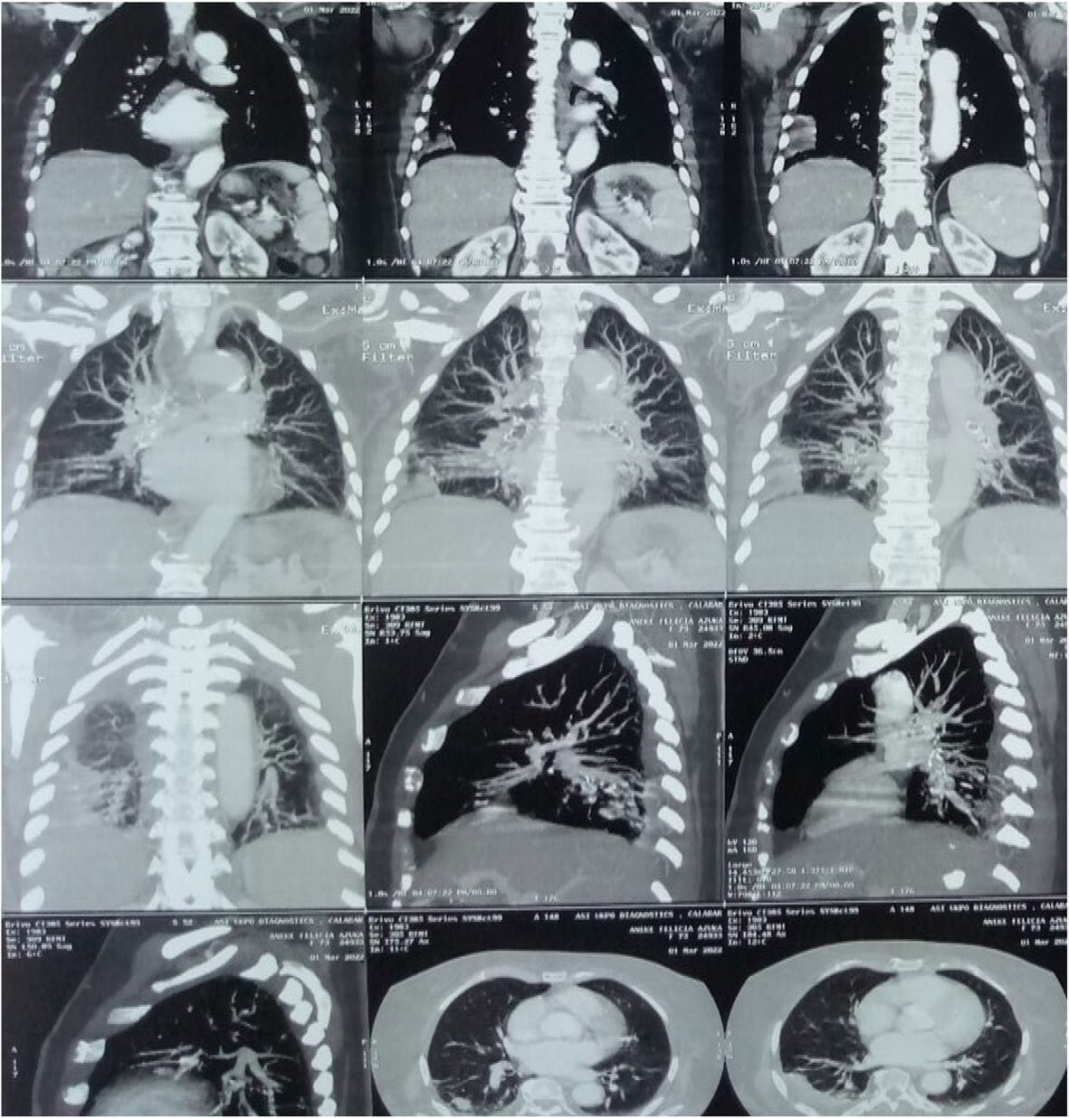
HRCT chest (contrast-enhanced) showing filling defects within the pulmonary arteries at the segmental and sub-segmental levels, pulmonary plethora, and right lower lobar consolidations

Computed tomography pulmonary angiography (CTPA) was requested, but due to its unavailability at the center as at then, contrast-enhanced high-resolution computed tomography (HRCT) of the chest was done, and it showed filling defects within the pulmonary arteries at the segmental and sub-segmental levels, which are features suggestive of PE (Fig. 3).

She was immediately started on subcutaneous enoxaparin 80 mg 12 hourly for 5 days. Her mental status improved over the next 3 days. She was then commenced on tablet dabigatran 110 mg twice daily which is to run for 6 months until we achieve a target INR of 1.2 to 1.8. The patient showed great recovery and at the third month outpatient follow-up, and she had achieved her target INR and remained fully orientated and lived independently at home.

## Discussion

Delirium, an acute confusional state, is a common, complex, potentially reversible, cognitive disturbance associated with substantial morbidity and mortality among patients of 65 years and above which is increasingly being recognized as a sign of serious underlying illness [13]. Elderly patients can experience delirium secondary to almost any acute condition. This includes simple conditions like untreated urinary tract infection, urinary retention, constipation, colds, and undermanaged pain, and more seriously, a variety of vascular conditions such as myocardial infarction, cerebral ischemia, and PE [10, 11].

Hypoxia is a well-known precipitating factor for delirium, and PE is a common cause of hypoxia. Delirium can complicate PE in patients with other features but occasionally may be the only finding in PE [11], as seen in our patient.

Similar reports have been documented in literature. Shaw et al. [10] reported two cases of acute confusional state secondary to episodes of hypoxia, resulting from repeated PE. Carrascosa et al. [11] described five elderly patients with delirium in whom PE was subsequently diagnosed and suggested a causal relationship (hypoxia) between both entities. Soysal et al. [14] also reported a case of hypoactive delirium caused by PE in an elderly adult. Similarly, Chen et al. [9] reported an atypical presentation of PE as acute confusion.

The initial diagnostic tests for PE include the D-dimer assay which is usually elevated in PE. It has good sensitivity, but poor specificity [15]. The D-dimer level in our patient was 1902.5 ng/mL (normal < 500 ng/ml) and this heightened our suspicion.

Pulmonary angiogram is the gold standard for diagnosing PE. Its limited availability and associated adverse effect have limited its use. Computed tomography pulmonary angiography (CTPA) has proved to be the best alternative with over 90% specificity and sensitivity in diagnosing pulmonary embolism in the main, lobar, and segmental pulmonary arteries [16] and will show a filling defect, upon contrast enhancement, in the pulmonary artery or any of its branches [17].

The non-availability of CTPA in hospitals in sub-Saharan Africa (including our center) is a constraint in the confirmation of most cases of PE [18].

Recently, contrast-enhanced CT of the chest has been found to be useful in the evaluation of pulmonary vascular diseases including PE [19–21], with some of the most recent studies reporting sensitivities of approximately 50% [22]. That was what we used in diagnosing PE in our patient whose image showed filling defects within the pulmonary arteries at the segmental and sub-segmental levels.

Other modalities for diagnosis of PE include ventilation-perfusion (V/Q) scanning (when CTPA is contraindicated), echocardiography which shows a nonspecific right ventricle (RV) strain, enlarged RV, tricuspid regurgitation, and sometimes, RV thrombi. Electrocardiography also shows sinus tachycardia as seen in our patient, deep *S* wave in lead 1, *Q* wave in lead 3, *T* wave inversion in lead 3 (S1Q3T3), tall *P* wave (*P* pulmonale), right bundle branch block, right ventricular hypertrophy, and right axis deviation [23]. Chest X-ray sometimes shows segmental atelectasis, pleural effusion, cardiomegaly, regional oligemia, enlarged pulmonary artery, and parenchymal opacities with unilateral diaphragmatic elevation [23]. This was seen in our patient (Fig. 1).

The treatment of PE in the acute phase with the use of heparin and other anticoagulants which prevent the propagation of old thrombi and the formation of new ones [23] has been a common practice. Following the use of anticoagulants, our patient’s cognitive function returned to normal within 3 days. A similar observation was made by Corrascora et al. [11]. Their five patients had a resolution of delirium in 2 to 5 days.

## Conclusion

Correct diagnosis and treatment of PE in elderly patients with delirium may improve mental function and increase their chances of full recovery and survival.

We therefore recommend that practitioners should include PE as a differential in elderly patients with delirium even in the absence of obvious risk factors. Such will enable them to initiate the necessary investigation and possibly start the necessary treatment, if confirmed, that will save a difficult situation.

## Data Availability

Not applicable.

## Abbreviations

CT: Computed tomography
CTPA: Computed tomography pulmonary angiography
℃: Degree centigrade
HRCT: High-resolution computed tomography
INR: International normalized ratio
MMSE: Mini: Mental State Examination
PE: Pulmonary embolism
